# Defending Our Public Biological Databases as a Global Critical Infrastructure

**DOI:** 10.3389/fbioe.2019.00058

**Published:** 2019-04-05

**Authors:** Jacob Caswell, Jason D. Gans, Nicholas Generous, Corey M. Hudson, Eric Merkley, Curtis Johnson, Christopher Oehmen, Kristin Omberg, Emilie Purvine, Karen Taylor, Christina L. Ting, Murray Wolinsky, Gary Xie

**Affiliations:** ^1^Sandia National Laboratories, Albuquerque, NM, United States; ^2^Los Alamos National Laboratory, Bioscience Division, Los Alamos, NM, United States; ^3^Los Alamos National Laboratory, Global Security Directorate, Los Alamos, NM, United States; ^4^Sandia National Laboratories, Livermore, CA, United States; ^5^Pacific Northwest National Laboratory, Richland, WA, United States

**Keywords:** cyberbiosecurity, biosecurity, cybersecurity, biological databases, machine learning, bioeconomy

## Abstract

Progress in modern biology is being driven, in part, by the large amounts of freely available data in public resources such as the International Nucleotide Sequence Database Collaboration (INSDC), the world's primary database of biological sequence (and related) information. INSDC and similar databases have dramatically increased the pace of fundamental biological discovery and enabled a host of innovative therapeutic, diagnostic, and forensic applications. However, as high-value, openly shared resources with a high degree of assumed trust, these repositories share compelling similarities to the early days of the Internet. Consequently, as public biological databases continue to increase in size and importance, we expect that they will face the same threats as undefended cyberspace. There is a unique opportunity, before a significant breach and loss of trust occurs, to ensure they evolve with quality and security as a design philosophy rather than costly “retrofitted” mitigations. This Perspective surveys some potential quality assurance and security weaknesses in existing open genomic and proteomic repositories, describes methods to mitigate the likelihood of both intentional and unintentional errors, and offers recommendations for risk mitigation based on lessons learned from cybersecurity.

## Introduction

Although an openly shared interaction platform confers great value to the biological research community, it may also introduce quality and security risks. Without a system for trusted correction and revision, these shared resources may facilitate widespread dissemination and use of low-quality content, for instance, taxonomically misclassified or erroneous sequences. Furthermore, as these public databases increase in size and importance, they may fall victim to the same security issues and abuses that plague cyberspace to this day. If we act now by developing the databases with quality and security as a design philosophy, we can protect these databases at a much lower cost and with fewer challenges than we currently face with the Internet.

In this Perspective, the authors aim to outline some potential quality assurance and security weaknesses in existing public biological repositories. In section Background: Problems With Public Biological Databases we provide a discussion of errors present in public biological databases and discuss possible security vulnerabilities inherent in their access, publication, and distribution models and systems. Both unintentional and intentional errors are discussed, the latter of which has not been given significant consideration in literature (Moussouni and Berti-Équille, 2013). In section Approaches for Improving Biological Databases, we attempt to introduce greater trust in the data and analyses by providing recommendations to mitigate or account for these errors and vulnerabilities and point to approaches used by other Internet databases. Finally, in section Preliminary Conclusions, we summarize our recommendations.

This Perspective focuses on databases which contain public and freely available data. We recognize that other biological databases exist which contain private, sensitive, or otherwise valuable data (e.g., human genomes). While unauthorized disclosure is not a formal concern in public, non-human databases, safeguarding against intentional or unintentional erroneous content is. Some approaches have been proposed to protect unauthorized disclosure (Kim and Lauter, 2015; Mandal et al., 2018; Ozercan et al., 2018) and, while we don't survey these approaches in this perspective, we note that the public database community may benefit from these ideas as well.

## Background: Problems With Public Biological Databases

### Data Integrity

An important goal for bioinformatics is the continuous improvement of biological databases. Given the rapid nature of this improvement and the rate of data production though, the content of these repositories is not without error. For example, the problem of contaminated sequences has been recognized for nearly two decades, with evidence stating that bacteria and human error are the two most common sources of contamination (Merchant et al., 2014; Strong et al., 2014). Ancient DNA is also particularly affected by human contamination (Pilli et al., 2013). These contaminants are frequently introduced during experiments (Merchant et al., 2014; Ballenghien et al., 2017) from natural associations and insufficient purification (Simion et al., 2017). In the past few years, additional reports have highlighted cases of DNA contamination in published genome data (Witt et al., 2009; Longo et al., 2011), suggesting that DNA contamination may be more widespread than previously thought. We recognize that errors and omissions can occur in open databases both at the sequence and at the metadata levels, but for this Perspective we mainly focus on sequence and taxonomic data concerns for the purposes of illustrating some of the many data integrity challenges possible.

In addition to contaminations, two high profile examples of sequence errors include the reassembly of a misassembled *Francisella tularensis* genome (Puiu and Salzberg, 2008) and the identification of single nucleotide errors in a reference *Tobacco mosaic virus* (TMV) genome (Cooper, 2014). Without a way to flag or remove the erroneous entries, future researchers are left to continually rediscover them. The errors in the *reference* TMV sequence are particularly disturbing. The taxonomic assignment corresponds to a pathogenic strain, but due to two erroneous single nucleotide polymorphisms (SNPs), virions synthesized from the published reference sequence are atypically *not* infectious. Overlooked contaminations in reference genomes can thereby lead to wrong or confusing results and may have major detrimental effects on biological conclusions (Philippe et al., 2011; Laurin-Lemay et al., 2012). While resequencing could be used to identify and correct sequence errors, it is only possible when the original source material is available. For the given example of single nucleotide errors in the TMV genome, the biological sample (sequenced in 1982) no longer exists. In addition to missing samples, samples of high consequence human and agricultural pathogens may not be available for resequencing.

Database integrity considerations for proteomics are generally similar to those for genomics because databases of protein sequences are derived from genome sequencing, via genome annotation and *in silico* translation. A sequence database error is unlikely to result in spurious detection of a protein that is present in the sample (false positive), but it could easily lead to a failure to detect a protein that is present (false negative). This is particularly concerning for discovery of accurate peptide signatures for use in targeted assays, a rapidly growing area of research.

In this section we discussed the issue of errors in genomic and proteomic databases and their impacts for research and application. Sources of these errors may include, among others, entry errors derived from data transfer, original errors derived from source data, and metadata errors (typically provenance-related) derived from the analysis pipeline. Original errors can arise from sequencing and sample preparation instrumentation chemistry, hardware, and software. Metadata errors can arise from bioinformatics software and faulty human interpretation. Each of these errors may be considered noise or the result of some other unintentional cause, but the key problem to note is that each element of the analytical process introduces some level of artifact when creating the analytical product, i.e., what is defined as a peak or a spot, what is the gene scaffold, what is the closed genome, etc. Any difference in process would therefore by its nature have some impact on the final genome. Our goal here is to start drawing connections between these process elements and genome anomalies.

### Vulnerabilities and Intentional Tampering

In contrast to the data integrity issues discussed in the prior section, errors may also be *intentionally* introduced into a biological database. For example, consider the hypothetical scenario discussed in Peccoud et al. (2018) whereby a graduate student reads an article and subsequently requests the plasmids described, but receives a faulty sample. It may be that the published sequences were fabricated, or that the source laboratory unwittingly sent faulty plasmids. One could also imagine a scenario where an intentionally mislabeled or harmful sequence is submitted to an open database that could later be unknowingly synthesized in a research setting or, more seriously, in a production capacity. Furthermore, depending on how sequences could be submitted to the database, the adversary may be able to keep the pathogenic sequence from being detected by certain anomaly detection heuristics.

Individuals may also exploit the vulnerabilities inherent in the database as a cyber-system, leading to errors introduced after publication of data despite manipulation and deletion controls. As with any database, biological databases can be compromised, enabling data integrity issues related to insertion, manipulation, exfiltration, and deletion of data, as well as providing a platform for privilege escalation, unauthorized surveillance, or distribution of malware. Ultimately, the effects of the operating environment and the tools used to deliver databases will inform the most appropriate threat model.

## Approaches for Improving Biological Databases

In 2000, a workshop titled *Bioinformatics: Converting Data to Knowledge* (National Research Council, 2000) tackled the question of biological database integrity as one of its focus areas. At that time, suggested solutions included building organism-type (e.g., eukaryote) specific grammar-based tools, enabling database self-validation through specialized ontologies, advocating for quality control in laboratories to minimize likelihood of errors, and authorizing only trained curators and annotators to enter data. They also recommend that data provenance be maintained so that the data history and evolution can be understood over time. These approaches fall more-or-less into two categories: ensuring integrity before or during data entry and analyzing data already in a database. Nearly 20 years later, we still emphasize the importance of quality control in laboratories and standardized data entry procedures, but it is clear that errors continue to make their way into databases for a variety of reasons. In this section, we highlight several categories of existing methods to detect data integrity issues in biological databases and outline the strengths and weaknesses of each. We also provide recommendations for improving biological database security.

### Automated Approaches for Detecting Anomalies

Some biological databases take the manual curation approach, such as the SwissProt subset of the UniProt (Universal Protein Resource Database). This effort requires significant resources to maintain, consisting of three principal investigators, a large staff and external advisory board (Pundir et al., 2017). Given the complexity and exponential growth of biological data, automatic methods are needed.

Some tools have been developed to assess the technical quality of genome assemblies [e.g., QUAST (Gurevich et al., 2013)], their completeness in terms of gene content [e.g., BUSCO (Simao et al., 2015), ProDeGe (Tennessen et al., 2016)] and even their contamination level [e.g., acdc (Lux et al., 2016), CheckM (Parks et al., 2015)]. Currently there are several analysis pipelines based on various searches to detect potentially contaminated sequences in the published and assembled genome, such as Taxoblast (Dittami and Corre, 2017), homology searches (Kryukov and Imanishi, 2016), GenomePeek (McNair and Edwards, 2015), and a multi-step cleaning process followed by a consensus of rankings (Cornet et al., 2018; Lu and Salzberg, 2018). All these tools require human review or use of additional tools to distinguish true positive from true negative and are therefore not feasible at scale.

Another database quality issue is the automated identification of taxonomically anomalous, questionable, or erroneous GenBank taxonomic assignments. Automated error identification of taxonomic assignments now draws on methods such as anomaly detection, classification, and prediction techniques. These methods have proved impactful in areas like computer vision (Krizhevsky et al., 2012) and natural language processing (Sutskever et al., 2014). They have also been adopted by bioinformatics and computational biology (Larranaga et al., 2006). Much of the work in applying machine learning to biological data is for classification and prediction of metadata, e.g., gene or taxonomy prediction in genomics, and structure and function prediction in proteomics. Verification of sequence metadata contained in a database is then performed by comparing with the predicted metadata from the sequence.

Sequence-based methods to detect taxonomically misclassified bacterial genome sequences tend to be based either on distance measures between pairs of sequences or on consistency with a reference 16S rRNA phylogeny. Common distance metrics include the average nucleotide identity (ANI), digital DNA-DNA hybridization (dDDH), multi-locus sequence analysis (MLSA), k-mer overlap (summarized in Federhen et al., 2016), and information theoretic distances (Li et al., 2004). Given a genome distance, taxonomic misclassifications have been discovered by identifying outlier genomes that exceed a manually determined distance threshold to trusted reference genomes (Goris et al., 2007; Colston et al., 2014; Figueras et al., 2014; Kim et al., 2014; Beaz-Hidalgo et al., 2015; Federhen et al., 2016; Tanizawa et al., 2016). The need for reference genomes is problematic, since approximately 20% of the bacterial genome sequences in GenBank currently (as of August, 2017) do not have a reference (or “type”) genome available (NCBI)^1^. The lack of bacterial genomes with a “type” designation is not due to the cost of sequencing, but rather the need to satisfy a specific set of formal requirements (Federhen, 2015), which include submitting culturable isolates to more than one culture collection. This poses a significant challenge for unculturable bacteria.

Distinct from these pairwise distance-based methods, a recent method for identifying taxonomically mislabeled sequences (Kozlov et al., 2016) uses consistency between a given set of taxonomic labels and a phylogenetic tree computed from a multiple sequence alignment of 16S rRNA sequences. This approach uses a single model of evolution to identify sequences whose taxonomic placement is most likely incorrect. However, there are multiple, competing methods for assigning bacterial taxonomy and, in particular, multiple sequence alignment of 16S rRNA can fail to resolve closely related species (Richter and Rossello-Mora, 2009; Kampfer and Glaeser, 2012;Larsen et al., 2014).

Machine learning has been applied to understand the sequences themselves. For example, the tools DeepBind (Alipanahi et al., 2015) and DeepSEA (Zhou and Troyanskaya, 2015) take sequences as input and learn how variations in the sequences can predict function. The successes of these tools coupled with recent research on sequence anomaly detection using long short-term memory (LSTM) recurrent neural networks (RNNs) in cyber security (Brown et al., 2018) could enable a new technique for biological sequence anomaly detection. Finally, if available, machine learning could potentially be applied to data concerning the sequence sources or data submitters themselves to evaluate quality and trustworthiness. However, that discussion is beyond the scope of this Perspective.

### Protections Against Intentional Errors

If a trusted method does not exist to ensure the continued quality and revision of content in biological databases, those who use the data should be aware of this risk and account for it in their analysis appropriately. In what follows, we outline previous efforts to develop analytics to detect and mitigate the impact of deliberately introduced database errors, both known and unknown.

Any machine learning analytic is necessarily a product of the data it observes. In an open data environment, an adversary can directly control any subsequent analysis by changing the data to change an algorithm's underlying model (Goodfellow et al., 2014). The focus of a “counter adversarial” approach to data analytics is to harden machine learning methods against the effects of inputs that are designed to mislead supervised (Dalvi et al., 2004; Kantarcioglu et al., 2011; Biggio et al., 2013a) and unsupervised (Dutrisac and Skillicorn, 2008; Biggio et al., 2013b) algorithms. It has been shown that there exist label tampering attacks which significantly decrease the accuracy of a classifier, while being nearly undetectable by standard cross validation tests (Kegelmeyer et al., 2015). In other words, the defender does not know the performance of the classifier has been corrupted. To protect against label tampering, an “ensembles of outlier measures” (EOM) method has been proposed to identify label tampering. The approach relies on a set of attributes that capture the “outlierness” of a sample to predict whether a sample has been tampered with. Tampered samples can then be remediated by changing the sample class label. In the context of a biological database, these labels may be metadata attributes associated with an entry. In the unsupervised machine learning scenario, an adversary may try to subvert a clustering algorithm by, for example, heuristically inserting data points to arbitrarily poison (i.e., merge) (Biggio et al., 2013b) clusters. In the context of a genomics database, poisoning of clusters would significantly reduce the ability to detect anomalous genomic sequences. Kegelmeyer et al. demonstrate that their remediation methodology based on an EOM applies equally well in the unsupervised context (Kegelmeyer et al., 2015).

As vulnerable cyber systems, best cyber practices can also be leveraged to protect biological databases. However, in the context of intentional manipulation of biological databases, special consideration must be given to the ability of these databases to enable production of dangerous biological material. The International Gene Synthesis Consortium (IGSC), for instance, provides two principal protections against the manufacture of malicious genetic material—known as the Harmonized Screening Protocol (International Gene Synthesis Consortium, 2017). The first, is a customer screening. The second is a screening of DNA sequences against a Regulated Pathogen Database (RPD). This database is built from data on the US Select Agent List, the Australian Group List, and other national lists of regulated pathogens. Members of the IGSC agree to translate each synthetic gene into amino acid sequence and test for homology. These are then accepted, reviewed or rejected. The RPD is updated annually and provided to members.

The Harmonized Screening Protocol requires at least two difficult processes—(1) sharing the database and (2) updating the database. Sharing the database requires the maintenance of authentication. Providers and users are part of a shared environment where they need to trust that everyone has an authentic and up-to-date version of the database. Updating the database requires maintenance to avoid “alert fatigue” from false positives and the dangerous potential case of false negatives resulting in malicious manufacture. Maintaining the security of this requires an environment of authentication and active database inspection and curation. For the former, there may be opportunities to incorporate advanced encryption and authentication algorithms being considered in the cyber domain such as blockchain. However, significant computational resource costs must be contended with.

## Preliminary Conclusions

This survey of concerns with biological databases and methods for ensuring database integrity is certainly not exhaustive but represents broad capabilities within data science and cybersecurity today that have shown promise either within computational biology already, or in tackling similar problems in other domains. A goal of the authors is to illuminate these concerns for a wide audience in the context of the historical lessons learned in cyberspace. In the early days of the Internet, the emphasis was on functionality and enabling the actions of largely well-intentioned communities of users. This functionality pervaded every element of our critical infrastructure. However, the same fabric that supports this infrastructure also represents a significant risk. Mitigating this risk after the wide penetration of open functionality is much more difficult than it might have been if the Internet had been created with integrity and security in mind. As biological data becomes a bedrock critical infrastructure for the entire bioeconomy and follows the same exponential trends of size, pervasiveness, and importance as the Internet, we have a unique opportunity to ensure that this capability mitigates current and future risks from a worldwide set of actors. This paper calls out several existing research areas that can be leveraged to protect against accidental and intentional modifications and misuse of public biological databases.

## Author Contributions

JC, JG, CH, EM, CO, EP, CT, and GX wrote sections of the manuscript. JC, JG, CH, EM, CJ, CO, KO, EP, KT, CT, and GX contributed to the formulation of the perspectives therein. NG, KT, and MW worked on document conception and assembly. All authors contributed to manuscript revision and approved of the submitted content.

### Conflict of Interest Statement

The authors declare that the research was conducted in the absence of any commercial or financial relationships that could be construed as a potential conflict of interest.

## Footnotes

^1^NCBI *Bacterial ANI Report* [Online]. Available: ftp://ftp.ncbi.nlm.nih.gov/genomes/ASSEMBLY_REPORTS/ANI_report_bacteria.txt (Accessed).
